# Leadership development in psychiatry: wellbeing, self-management, and mentorship across the early career stages

**DOI:** 10.1192/j.eurpsy.2026.10257

**Published:** 2026-07-31

**Authors:** G. Longo

**Affiliations:** Department of Clinical Neurosciences/DIMSC, Polytechnic University of Marche, Unit of Clinical Psychiatry, Ancona, Italy

## Abstract

**Abstract:**

Leadership has become an essential competency in contemporary psychiatry, where clinicians are required to navigate increasing clinical complexity, multidisciplinary teamwork, and evolving healthcare systems. However, leadership development is often conceptualized primarily in terms of managerial or organizational skills, with limited attention to the role of personal wellbeing and self-management. This presentation proposes a wellbeing-centred model of leadership development in psychiatry, in which effective leadership begins with self-management capacities, including awareness of personal limits, prioritization, time management, and emotional regulation. Mentoring relationships can foster reflective learning, reduce professional isolation, and promote sustainable career trajectories. Within this perspective, wellbeing is reframed not as an individual achievement but as a prerequisite for effective and sustainable leadership in psychiatry.

**Disclosure of Interest:**

None Declared

